# Notes and Notices

**Published:** 1896-12

**Authors:** 


### NOTES AND NOTICES.

Dr. J. H. Allen, formerly of Logansport, Indiana, has removed to 186 Thirty-seventh Street, Chicago, where he will continue the practice of Hahnemannian Homœopathy. Dr. Allen has been appointed Professor of Skin and Venereal Diseases in Hering Medical College.

Notice.—Owing to the crowded condition of our pages this month, and the addition of the year's index, it is necessary to omit the usual complement of pages of Hering on Typhoid Fever. The late appearance of the journal each month is unavoidable, as the editor has no assistants, and must, in addition, attend to a large practice.

